# The virulence contribution of the CFEM family genes of *Beauveria bassiana* is closely influenced by the external iron environment

**DOI:** 10.1128/spectrum.03096-24

**Published:** 2025-03-21

**Authors:** Xu Zhang, Guang Wang, Bin Chen, YueJin Peng

**Affiliations:** 1 Collage of Plant Protection in Yunnan Agricultural University, Kunming, Yunnan, China; Institute of Microbiology, Chinese Academy of Sciences, Beijing, China

**Keywords:** CFEM gene family, *Beauveria bassiana*, iron starvation regulation, infection cycle, compensation mechanism

## Abstract

The common in fungal extracellular membrane (CFEM) domain proteins represent characteristic fungal extracellular membrane proteins. The entomopathogenic fungus *Beauveria bassiana* contains 12 CFEM domain proteins involved in the generation of iron hunger response. However, many *BbCFEM* genes that infect insects do not promote fungal virulence. In this study, we systematically assessed the role of the *BbCFEM* family in fungal virulence under moderate iron concentrations and severe iron starvation (0.4 mM BPS) induced by body wall infection and injection infection. The results showed that the *BbCFEM* family members have different functions based on virulence, which is directly affected by external iron levels. Quantitative real-time reverse-transcription polymerase chain reaction preliminarily demonstrated that deletion of *BbCFEM* family genes significantly increased the expression levels of other family members. The increased expression levels compensated for the gene damage induced by the deletion of *BbMFEM* genes. In addition, the results showed that the *BbCFEM* gene participates in the initiation of fungal responses to cell wall stress and oxidative stress. These findings reveal the evolutionary strategies employed by pathogenic fungi to adapt to the environment and population reproduction. This study expands our understanding of the mechanism of this gene family of entomopathogenic fungi in infecting pests.

**IMPORTANCE:**

The common in fungal extracellular membrane (CFEM) domain is a fungal extracellular membrane protein that can trap heme, which assists in fungal infection and colonization. *Beauveria bassiana* is an entomopathogenic fungus that is widely used to control pests. We systematically assessed the contribution of the *BbCFEM* family to *B. bassiana*’s virulence under severe iron starvation and *B. bassiana*’s growth and stress resistance under moderate iron levels. We found that the *BbCFEM* family members have different functions based on virulence with severe iron starvation, which also plays an important role in fungal responses to cell wall stress and oxidative stress. This study provides new insights into the genetic families of entomopathogenic fungi and the mechanisms by which they infect pests.

## INTRODUCTION

*Beauveria bassiana* is an entomopathogenic fungus that is widely used to control pests (1). *B. bassiana* conidia induce tube germination when they come into contact with an arthropod host by applying mechanical pressure while secreting various body surface degrading enzymes that help the fungus enter the arthropod host (2). Next, the infectious mycelium penetrates the body surface and enters the insect’s blood cavity through dimorphic transformation. As a result, an internal mycelium develops in the insect’s blood cavity (3, 4). The mycelium in the saprophytic state penetrates the body surface of the host following the death of the host by fungal infection and covers the whole body, producing several conidia, thus entering a new infection cycle (5).

It is well known that fungi have a rigorous iron acquisition system, the core of which is a negative feedback regulatory system formed by bZIP type transcription factor Hap and GATA type transcription factor Sre (6). Taken together, they regulate the iron balance in the fungus, keeping iron levels at a stable level. In addition, fungi also have reduced iron assimilation, Fe-carrier regulated Fe^3+^ absorption, and fe-heme absorption systems (7, 8). The common in fungal extracellular membrane (CFEM) domain is a component of fungal extracellular membrane proteins that contains eight conserved cysteine residues that can trap heme. CFEM has been recently studied in many fungi, including insect entomopathogenic fungi (9, 10), plant pathogenic fungi (11–13), and human pathogenic fungi (14, 15). Several pathology and physiology studies have also revealed the importance of CFEM domain proteins for the overall development and pathogenicity of fungi. For example, the CFEM domain of Pth11, which is required for the development and pathogenicity of fungi (16), is responsible for hydrophobicity, infection, surface sensing, and hyper-conidiation in *Magnaporthe oryzae* (17). The CFEM protein of *Candida albicans* maintains cell wall structure (18, 19). CFEM domain-containing protein of *Saccharomyces cerevisiae* (Ccw14) and *Aspergillus fumigatus* CFEM-domain GPI-anchored proteins (CfmA-C) also play key roles in cell wall structure (15, 20). Notably, CfmA-C of *A. fumigatus* does not promote fungal virulence (15). Although the roles of CFEM proteins in general, the functional differentiation and compensation in *BbCFEM* genes under varying iron conditions remain unknown.

The motifs of the CFEM domain can be summarized as: PxC[A/G]x_2_Cx_8–12_Cx_1–3_[x/T]Dx_2–5_CxCx_9–14_Cx_3–4_Cx_15–16_C (21). Studies have shown that CFEM domain proteins play an important role in heme-iron trapping in fungi, thus assisting in fungal infection and colonization. For example, a semi-conserved aspartic acid exists between the third and fourth cysteines (22), which is also closely related to the capture of heme iron by CFEM. The CFEM proteins in *C. albicans* (Rbt5, Pga7, and Csa2) can obtain heme from host hemoglobin, thereby mediating its transfer to fungal cells (23–25). The same was true in *Candida parapsilosis* (26) and *Cryptococcus neoformans*, thereby supplementing the iron acquisition pathway (27). In addition, for capturing iron ions, Peng et al. identified 12 CFEM domain proteins from *B. bassiana* filamentous entomopathogenic fungi and genetically characterized the virulence contributions of 11 of these genes from two forms of iron, including heme and iron ions (28).

Many genetic characterization and pathological analyses of the CFEM family of fungi have shown that some genes have definite functions, while other genes have very weak or no roles. For example, transcriptome analysis of genes encoding CFEM in *Fusarium graminis* showed that all 23 *CFEM* genes are expressed during wheat infection, where 7 genes are significantly upregulated and 6 genes are significantly downregulated (29). However, further studies should verify the function of the remaining 10 genes. Similarly, *BbCFEM7* and *8* significantly infect insect hosts in *B. bassiana* (28). The contribution of the other nine genes to fungal virulence is still unknown.

This study hypothesizes that *BbCFEM* genes exhibit differential contributions to fungal virulence and stress tolerance depending on external iron availability. Based on that hypothesis, we systematically characterized the virulence of wild strains of *B. bassiana* and 11 knockout strains of the CFEM family infected with *Galleria mellonella* by creating a severe iron starvation environment. The results revealed that the virulence of fungi contributed by 11 genes of the *BbCFEM* family under severe iron-starved conditions was different from that contributed under moderate iron-starved conditions (28). Moreover, for the first time, the division of labor within the CFEM family under varying iron starvation conditions was reviewed, and a hypothesis was presented regarding the functional compensation of *BbCFEM* family genes during host infection. Therefore, this study provides a new direction for the systematic analysis of fungal *CFEM* domain gene function and a theoretical basis for virulence analysis of entomopathogenic fungi in different environments. More importantly, the improvement of iron acquisition mechanisms of *CFEM* family genes is helpful for the application of biocontrol fungi in pest control.

## MATERIALS AND METHODS

### Microbial strains and cultivation

The wild-type (WT) strain of *B. bassiana* ARSEF2860 was purchased from the U.S. Department of Agriculture Entomopathogenic Fungus Collection (Ithaca, NY, USA). The mutant strains of Δ*BbCFEM1*–Δ*BbCFEM11* were developed as previously described (28). WT and mutation strains were maintained in the SDAY plates (4% glucose, 1% peptone, 1% yeast extract, and 1.5% agarose) at 25°C. Czapek-Dox agar (CZA) (3% glucose, 0.3% NaNO_3_, 0.1% K_2_HPO_4_, 0.05% KCl, 0.05% MgSO_4_, 0.001% FeSO_4_, and 1.5% agar) was used as the chemically defined medium.

### Assays for fungal growth and stress responses

Fungal phenotypes of the WT and mutant strains were examined as previously described (28). All assays were repeated three times. The vegetative growth was evaluated based on various carbon (3% glucose, 3% sucrose, 3% trehalose, 3% galactose, 3% maltose, 3% lactose, and 3% mannose) and nitrogen sources (0.3% NaNO_2_, 3% NH_4_Cl, 3% (NH_4_)_2_SO_4_, 3% N-GluNAc, 3% gelatin, and 3% chitin) with CZA medium. Fungal growth under stress was investigated using the CZA plates with 0.02 mM menadione, 2 mM H_2_O_2_, 1.2 M sorbitol, 0.5 M NaCl, 6 µg/mL Congo red, and 3 mM Zn^2+^. The conidia grown on SDAY plates were suspended in 0.02% Tween-80 at the final concentration of 10^6^ /mL. Aliquots of the suspension (1 µL) were inoculated on the indicated plates, and colony diameters were measured at 7 days post-incubation at 25°C.

### Fungal virulence under iron-starved conditions

Conidial virulence was evaluated using *G. mellonella* larvae as the bioassay hosts (28). Conidia of the indicated strains were harvested after 8 days of culture on SDAY plates. For topical infection, the larvae were immersed in conidial suspensions (10^7^ /mL) for 15 s, with 0.02% Tween-80 as blank control. Furthermore, 5 µL of a 10^5^ conidia/mL suspension was injected into the host hemocoel for intrahemocoel infection assay. Bathophenanthroline disulfonate (0.4 mM, BPS) was added to control iron level in the conidial suspension. Each treatment had three replicates, with 30–40 insects in each replicate. Mortality was recorded every 12 h from 3 to 6 days post-infection (dpi), then every 24 h till 10 dpi. The median lethal time (LT_50_) values were calculated for each strain via the Kaplan–Meier method. The differences between the paired curves for each strain were calculated using the log-rank test.

### Quantitative real-time reverse-transcription polymerase chain reaction (RT-qPCR)

Aliquots (100 µL) of conidial suspension (1 × 10^6^ conidia/mL) were smeared onto SDAY plates covered with glass paper and incubated at 25°C for 3 days to determine gene expression. Total RNA was isolated using RNAiso Plus Reagent (TaKaRa, Dalian, China) following the manufacturer’s instructions. A Prime Script RT reagent Kit Perfect Real Time for qPCR (TaKaRa) was used to synthesize the first-strand cDNA from total RNA. qPCR analyses with paired primers were performed using SYBR Premix Ex Taq (TaKaRa). The transcript of the fungal *β-actin* gene was used as an internal standard. The relative expression of every gene was determined via the 2^−ΔΔCT^ technique. The primers utilized in qPCR tests were shown in Table S1.

### Statistical analyses

All statistical analyses were performed using GraphPad Prism 8 (GraphPad Software, Inc.). The data were presented as the mean ± standard deviation. Unpaired Student’s *t*-test and one-way analysis of variance followed by a Tukey’s *post hoc* test were used to compare between two groups and among multiple groups, respectively. *P* < 0.05 was considered statistically significant.

## RESULTS

### Effects of *BbCFEM* genes on fungal growth

The effects of *BbCFEM* gene loss on fungal vegetative growth were examined using various carbon and nitrogen sources. The growth defect of Δ*BbCFEM7* and Δ*BbCFEM8* strains on glucose, trehalose, maltose, galactose, lactose, and sucrose, with control (WT and individual complemented strain) was shown in Table 1. The Δ*BbCFEM1*, *6*, *7*, *8*, *9*, and *11* strains had significantly decreased vegetative growth on mannose compared to the control. Notably, colony diameter was not significantly different among mutant, WT, and individual complemented strains. Similar results were also observed on the indicated nitrogen sources. The Δ*BbCFEM7* and Δ*BbCFEM8* strains had a significantly decreased colony diameter on nitrogen sources (Table 2), while other mutation strains showed no growth defects during incubation on CZA amended with all nitrogen sources. These results suggested that *BbCFEM7* and *BbCFEM8* genes influenced the growth of *B. bassiana*, which exerted the most significant effect on fungal growth in response to carbon and nitrogen sources.

**TABLE 1:** Phenotypic comparison of vegetative growth (carbon source) between the wild-type and mutant strains^*a*^

	Glucose	Trehalose	Maltose	Galactose	Lactose	Mannose	Sucrose
WT	1.32 ± 0.04	1.47 ± 0.05	1.20 ± 0.03	1.26 ± 0.03	1.41 ± 0.01	1.21 ± 0.02	1.43 ± 0.03
Δ1	1.25 ± 0.03	1.49 ± 0.02	1.21 ± 0.02	1.22 ± 0.01	1.35 ± 0.05	1.06 ± 0.05**	1.45 ± 0.05
Δ1Com	1.27 ± 0.05	1.50 ± 0.00	1.28 ± 0.04	1.21 ± 0.01	1.40 ± 0.01	1.10 ± 0.01	1.41 ± 0.01
Δ2	1.23 ± 0.06	1.50 ± 0.02	1.14 ± 0.12	1.21 ± 0.02	1.37 ± 0.06	1.07 ± 0.06	1.40 ± 0.06
Δ2Com	1.34 ± 0.02	1.50 ± 0.01	1.28 ± 0.07	1.20 ± 0.01	1.40 ± 0.02	1.07 ± 0.06	1.41 ± 0.02
Δ3	1.36 ± 0.01	1.50 ± 0.04	1.20 ± 0.02	1.20 ± 0.02	1.29 ± 0.02	1.11 ± 0.01	1.47 ± 0.04
Δ3Com	1.31 ± 0.06	1.50 ± 0.01	1.21 ± 0.01	1.20 ± 0.01	1.41 ± 0.03	1.14 ± 0.05	1.50 ± 0.05
Δ4	1.31 ± 0.01	1.50 ± 0.02	1.21 ± 0.02	1.21 ± 0.05	1.30 ± 0.01	1.10 ± 0.01	1.43 ± 0.06
Δ4Com	1.30 ± 0.01	1.51 ± 0.01	1.20 ± 0.03	1.21 ± 0.01	1.41 ± 0.02	1.18 ± 0.04	1.42 ± 0.04
Δ5	1.27 ± 0.06	1.42 ± 0.05	1.18 ± 0.03	1.20 ± 0.00	1.40 ± 0.02	1.07 ± 0.12	1.40 ± 0.03
Δ5Com	1.30 ± 0.01	1.50 ± 0.00	1.27 ± 0.06	1.19 ± 0.02	1.39 ± 0.01	1.10 ± 0.02	1.41 ± 0.02
Δ6	1.23 ± 0.06	1.43 ± 0.06	1.20 ± 0.01	1.21 ± 0.01	1.33 ± 0.06	1.03 ± 0.06**	1.41 ± 0.06
Δ6Com	1.30 ± 0.00	1.51 ± 0.02	1.27 ± 0.06	1.20 ± 0.00	1.42 ± 0.033	1.11 ± 0.01	1.40 ± 0.01
Δ7	0.90 ± 0.01***	1.01 ± 0.01***	0.70 ± 0.00***	0.80 ± 0.00***	0.81 ± 0.01***	0.71 ± 0.02***	1.01 ± 0.01
Δ7Com	1.31 ± 0.02	1.49 ± 0.04	1.27 ± 0.05	1.21 ± 0.01	1.41 ± 0.05	1.11 ± 0.02	1.4 ± 0.02
Δ8	1.00 ± 0.03***	1.11 ± 0.01***	0.71 ± 0.02***	0.83 ± 0.06***	0.84 ± 0.06***	0.71 ± 0.01***	1.01 ± 0.01
Δ8Com	1.31 ± 0.01	1.50 ± 0.03	1.28 ± 0.05	1.21 ± 0.01	1.41 ± 0.02	1.11 ± 0.01	1.40 ± 0.02
Δ9	1.26 ± 0.05	1.44 ± 0.02	1.21 ± 0.12	1.22 ± 0.04	1.35 ± 0.03	1.06 ± 0.04**	1.43 ± 0.03
Δ9Com	1.31 ± 0.01	1.51 ± 0.01	1.27 ± 0.06	1.20 ± 0.01	1.43 ± 0.06	1.12 ± 0.01	1.41 ± 0.04
Δ10	1.34 ± 0.02	1.53 ± 0.02	1.21 ± 0.02	1.27 ± 0.02	1.32 ± 0.05	1.10 ± 0.01	1.43 ± 0.02
Δ10Com	1.31 ± 0.01	1.51 ± 0.02	1.20 ± 0.12	1.21 ± 0.01	1.41 ± 0.02	1.18 ± 0.04	1.43 ± 0.06
Δ11	1.23 ± 0.06	1.43 ± 0.06	1.23 ± 0.01	1.26 ± 0.02	1.33 ± 0.06	1.03 ± 0.06**	1.41 ± 0.01
Δ11Com	1.31 ± 0.03	1.50 ± 0.01	1.27 ± 0.06	1.21 ± 0.01	1.41 ± 0.01	1.10 ± 0.01	1.41 0.01

^*a*^ Comparison of the indicated phenotype between the wild-type and mutant strain was subjected to Student’s *t*-test. **, *P* < 0.01; ***, *P* < 0.001.

**TABLE 2:** Phenotypic comparison of vegetative growth (nitrogen source) between the wild-type and mutant strains^*a*^^,^^*b*^^,*c*^

	NaNO_2_	NH_4_Cl	(NH_4_)_2_SO_4_	N-GluNAc	Gelatin	Chitin
WT	1.47 ± 0.06	1.63 ± 0.06	1.63 ± 0.06	1.50 ± 0.05	2.22 ± 0.02	1.52 ± 0.04
Δ1	1.51 ± 0.01	1.70 ± 0.03	1.63 ± 0.06	1.50 ± 0.03	2.22 ± 0.02	1.47 ± 0.06
Δ1Com	1.51 ± 0.06	1.81 ± 0.03	1.61 ± 0.01	1.57 ± 0.06	2.21 ± 0.03	1.51 ± 0.04
Δ2	1.51 ± 0.02	1.72 ± 0.02	1.63 ± 0.06	1.50 ± 0.06	2.20 ± 0.12	1.50 ± 0.01
Δ2Com	1.42 ± 0.02	1.70 ± 0.01	1.63 ± 0.06	1.52 ± 0.02	2.21 ± 0.02	1.62 ± 0.05
Δ3	1.43 ± 0.06	1.70 ± 0.03	1.72 ± 0.02	1.50 ± 0.03	2.23 ± 0.06	1.47 ± 0.06
Δ3Com	1.47 ± 0.06	1.69 ± 0.01	1.71 ± 0.03	1.51 ± 0.01	2.19 ± 0.04	1.49 ± 0.03
Δ4	1.44 ± 0.03	1.73 ± 0.00	1.72 ± 0.02	1.51 ± 0.03	2.23 ± 0.06	1.52 ± 0.03
Δ4Com	1.47 ± 0.02	1.71 ± 0.01	1.71 ± 0.04	1.52 ± 0.02	2.23 ± 0.05	1.57 ± 0.06
Δ5	1.46 ± 0.03	1.69 ± 0.04	1.63 ± 0.06	1.47 ± 0.06	2.17 ± 0.06	1.53 ± 0.06
Δ5Com	1.40 ± 0.01	1.72 ± 0.03	1.62 ± 0.03	1.52 ± 0.03	2.21 ± 0.01	1.58 ± 0.03
Δ6	1.43 ± 0.06	1.71 ± 0.04	1.63 ± 0.06	1.51 ± 0.01	2.19 ± 0.06	1.51 ± 0.01
Δ6Com	1.50 ± 0.05	1.82 ± 0.03***	1.63 ± 0.06	1.50 ± 0.12	2.17 ± 0.06	1.52 ± 0.04
Δ7	1.03 ± 0.06***	1.32 ± 0.03***	1.11 ± 0.01***	1.03 ± 0.06***	2.01 ± 0.01***	1.71 ± 0.05***
Δ7Com	1.43 ± 0.06	1.71 ± 0.02	1.61 ± 0.02	1.51 ± 0.02	2.21 ± 0.01	1.58 ± 0.03
Δ8	1.03 ± 0.12***	1.41 ± 0.06***	1.23 ± 0.06***	1.00 ± 0.03***	1.82 ± 0.02***	1.61 ± 0.02
Δ8Com	1.43 ± 0.12	1.72 ± 0.06	1.61 ± 0.02	1.48 ± 0.03	2.21 ± 0.02	1.62 ± 0.03
Δ9	1.44 ± 0.21	1.73 ± 0.06	1.69 ± 0.09	1.50 ± 0.06	2.23 ± 0.06	1.52 ± 0.03
Δ9Com	1.47 ± 0.06	1.67 ± 0.06	1.70 ± 0.02	1.51 ± 0.03	2.27 ± 0.06	1.57 ± 0.06
Δ10	1.43 ± 0.20	1.71 ± 0.01	1.73 ± 0.05	1.52 ± 0.03	2.23 ± 0.06	1.47 ± 0.06
Δ10Com	1.43 ± 0.03	1.73 ± 0.06	1.67 ± 0.06	1.52 ± 0.03	2.18 ± 0.03	1.49 ± 0.04
Δ11	1.47 ± 0.02	1.72 ± 0.03	1.73 ± 0.05	1.47 ± 0.06	2.23 ± 0.06	1.47 ± 0.06
Δ11Com	1.48 ± 0.03	1.70 ± 0.00	1.73 ± 0.06	1.49 ± 0.01	2.21 ± 0.02	1.50 ± 0.03

^*a*^ Vegetative growth was evaluated on different carbon (C) and nitrogen (N) sources, using SDAY as the control of rich medium.

^*b*^ Δ1–11, the gene disruptants ΔBbCFEM1–11. Com, the complemented strain.

^*c*^ Comparison of the indicated phenotype between the wild-type and mutant strain was subjected to Student’s *t*-test. ***, *P* < 0.001.

### Effects of *BbCFEM* genes on multiple stress responses

Chemical stress is an important factor affecting fungal adaptability. The response of *BbCFEM* family genes to several chemical stress agents was systematically tested. Almost all mutant strains demonstrated a considerable increase in sensitivity to Congo red stress (Table 3), except for Δ*BbCFEM9*. Furthermore, Δ*BbCFEM6*, *7*, *8*, and *11* strains showed an enhanced sensitivity to Zn^2+^ stress. Compared to the WT and complemented strains, the *BbCFEM7*, *8*, and *11* mutants showed significant sensitivity to sorbitol stress. Moreover, the *BbCFEM* family gene mutants showed a similar overall trend when exposed to NaCl, menadione, and H_2_O_2_. These findings indicate that disruption of *BbCFEM* genes results in different effects on *B. bassiana* growth under multiple stresses.

**TABLE 3:** Phenotypic comparison of stress chemicals between the wild-type and mutant strains^*a*^^*,^b^,*^^*c*^

	Congo red	Zn^**2+**^	Sorbitol	NaCl	Menadione	H_**2**_O_**2**_
WT	1.51 ± 0.06	2.14 ± 0.01	1.53 ± 0.06	1.50 ± 0.03	1.53 ± 0.06	1.51 ± 0.04
Δ1	1.33 ± 0.02***	2.11 ± 0.06	1.50 ± 0.01	1.31 ± 0.01***	1.54 ± 0.01	1.24 ± 0.02***
Δ1Com	1.54 ± 0.02	2.13 ± 0.01	1.50 ± 0.12	1.53 ± 0.05	1.52 ± 0.02	1.50 ± 0.02
Δ2	1.32 ± 0.06***	2.10 ± 0.02	1.50 ± 0.06	1.32 ± 0.00***	1.52 ± 0.06	1.22 ± 0.00***
Δ2Com	1.43 ± 0.12*	2.10 ± 0.01	1.50 ± 0.05	1.43 ± 0.12*	1.51 ± 0.06	1.52 ± 0.01
Δ3	1.32 ± 0.02***	2.13 ± 0.02	1.53 ± 0.02	1.34 ± 0.06***	0.93 ± 0.06***	1.10 ± 0.04***
Δ3Com	1.52 ± 0.01	2.12 ± 0.02	1.51 ± 0.01	1.52 ± 0.04	1.50 ± 0.05	1.50 ± 0.02
Δ4	1.20 ± 0.00***	2.12 ± 0.03	1.50 ± 0.04	1.52 ± 0.02	1.33 ± 0.00***	1.04 ± 0.04***
Δ4Com	1.50 ± 0.05	2.13 ± 0.06	1.53 ± 0.01	1.51 ± 0.12	1.50 ± 0.06	1.50 ± 0.06
Δ5	1.30 ± 0.01***	2.13 ± 0.06	1.50 ± 0.03	1.40 ± 0.01***	1.00 ± 0.01***	1.24 ± 0.05***
Δ5Com	1.50 ± 0.02	2.10 ± 0.01	1.53 ± 0.06	1.52 ± 0.02	1.50 ± 0.02	1.52 ± 0.06
Δ6	1.34 ± 0.12***	1.90 ± 0.00***	1.52 ± 0.00	1.42 ± 0.06***	0.83 ± 0.06***	1.32 ± 0.01***
Δ6Com	1.51 ± 0.05	2.12 ± 0.12	1.54 ± 0.01	1.52 ± 0.06	1.50 ± 0.12	1.51 ± 0.060
Δ7	0.50 ± 0.04***	1.40 ± 0.20***	0.52 ± 0.00***	0.77 ± 0.06***	0.63 ± 0.00***	1.50 ± 0.01
Δ7Com	1.54 ± 0.00	2.13 ± 0.01	1.53 ± 0.06	1.50 ± 0.05	1.52 ± 0.02	1.51 ± 0.02
Δ8	0.40 ± 0.02***	1.41 ± 0.02***	0.50 ± 0.04***	0.74 ± 0.12***	0.43 ± 0.06***	1.50 ± 0.06
Δ8Com	1.53 ± 0.02	2.10 ± 0.05	1.50 ± 0.01	1.54 ± 0.02	1.54 ± 0.04	1.50 ± 0.01
Δ9	1.51 ± 0.02	2.13 ± 0.06	1.50 ± 0.02	1.52 ± 0.02	1.52 ± 0.02	1.52 ± 0.06
Δ9Com	1.52 ± 0.12	2.13 ± 0.06	1.51 ± 0.06	1.50 ± 0.01	1.52 ± 0.03	1.50 ± 0.05
Δ10	1.33 ± 0.04***	2.14 ± 0.04	1.54 ± 0.06	1.30 ± 0.06***	1.33 ± 0.06***	1.31 ± 0.00***
Δ10Com	1.50 ± 0.01	2.14 ± 0.01	1.54 ± 0.12	1.51 ± 0.02	1.54 ± 0.02	1.50 ± 0.01
Δ11	1.03 ± 0.06***	1.53 ± 0.00***	1.40 ± 0.02***	1.40 ± 0.01***	1.20 ± 0.01***	1.04 ± 0.03***
Δ11Com	1.54 ± 0.00	2.13 ± 0.06	1.50 ± 0.06	1.50 ± 0.02	1.50 ± 0.05	1.50 ± 0.02

^*a*^ Vegetative growth was evaluated on different carbon (C) and nitrogen (N) sources, using SDAY as the control of rich medium.

^*b*^ Δ1–11, the gene disruptants ΔBbCFEM1–11. Com, the complemented strain.

^*c*^ Comparison of the indicated phenotype between the wild-type and mutant strain was subjected to Student’s *t*-test. *, *P* < 0.05; **, *P* < 0.01; ***, *P* < 0.001.

### *BbCFEM* gene was required for fungi proliferation under iron starvation

**Fig 1:**
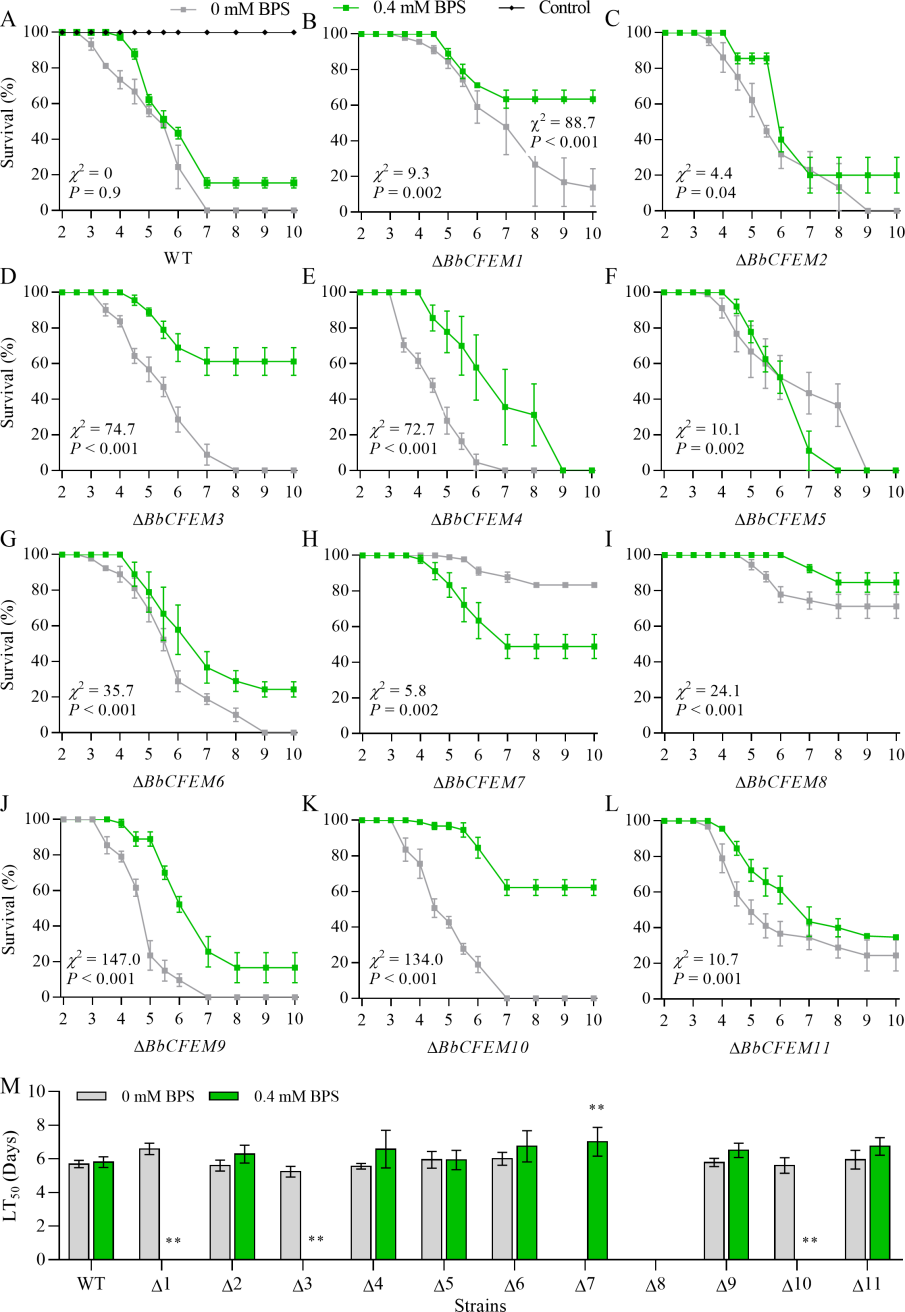
Virulence contribution of the *BbCFEM* family genes under iron starvation conditions through topical infection. (A–L) Survival curves of topical infection insects with the conidial suspension (10^7^ conidia mL^−1^) of wild-type strain and Δ*BbCFEM1–11* mutant strains under 0 mM BPS and 0.4 mM BPS, respectively. (M) The lethal time (LT_50_) between topical infection of insect larvae with wild-type strains and Δ*BbCFEM1–11* mutants under 0 mM BPS and 0.4 mM BPS.

**Fig 2:**
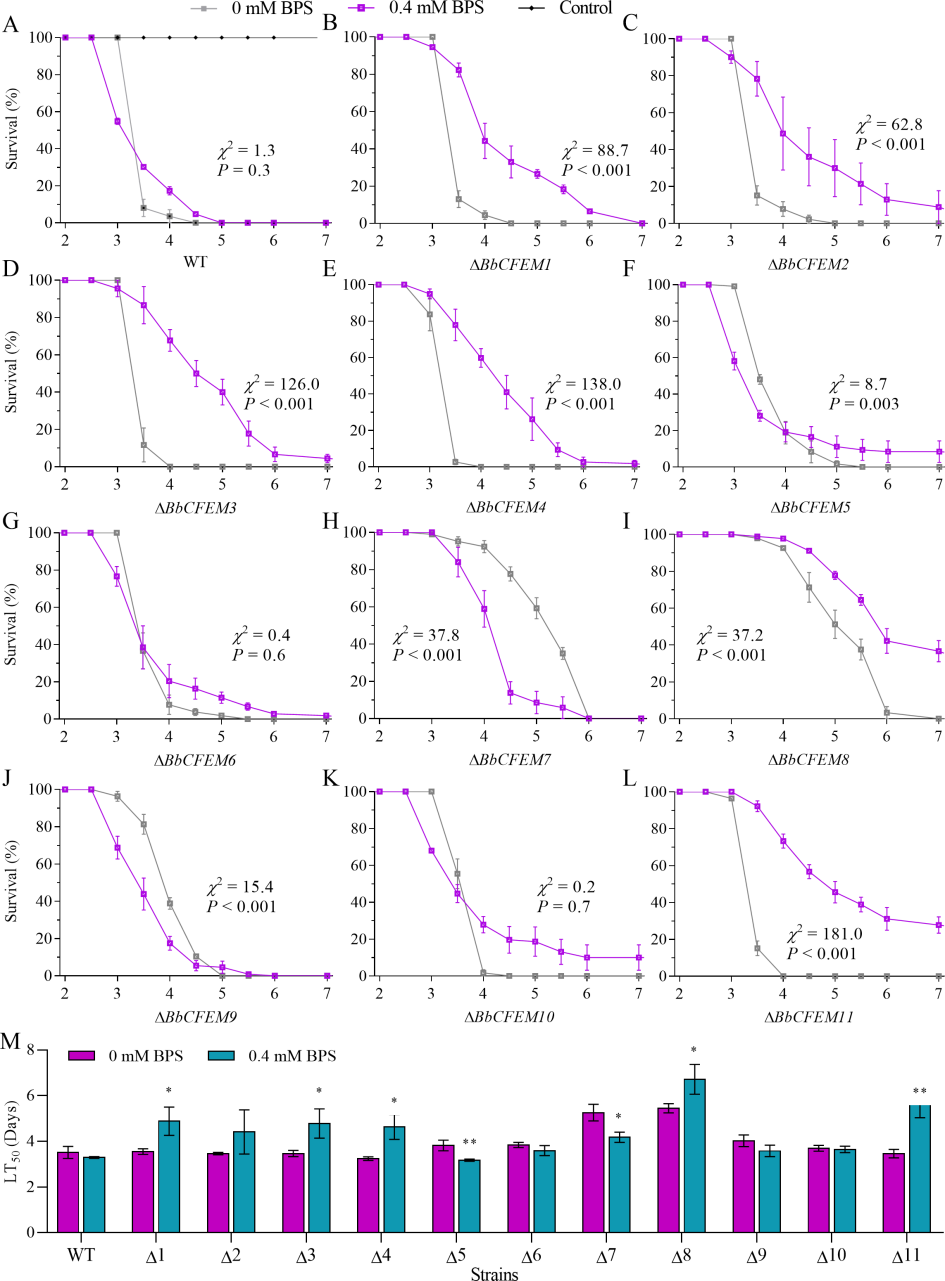
Virulence contribution of the *BbCFEM* family genes under iron starvation conditions through injection infection. (A–L) Survival curves of injection infection insect with the conidial suspension (5 µL, 10^5^ conidia mL^−1^) of wild-type strain and Δ*BbCFEM1–11* mutant strains under 0 mM BPS and 0.4 mM BPS, respectively. (M) The lethal time (LT_50_) between injection infection insect larvae with wild-type strains and Δ*BbCFEM1–11* mutants under 0 mM BPS and 0.4 mM BPS.

A previous study reported that *BbCFEM* genes contribute to the virulence of *B. bassiana*. Herein, the survival rate of *G. mellonella* was analyzed to explore the role of iron in the pathogenicity of *BbCFEM* genes. The virulence of *BbCFEM* gene loss strains was significantly affected in both topical infection and injection infection when the iron chelator BPS (0.4 mM) was added to the conidial suspension to induce iron starvation (Fig. 1 and 2; Table 4). Moreover, iron starvation did not affect the survival curve of the WT strain in the topical infection (Fig. 1A), but significantly reduced the virulence of Δ*BbCFEM1* (Fig. 1B), *2* (Fig. 1C), *3* (Fig. 1D), *4* (Fig. 1E), *6* (Fig. 1G), *8* (Fig. 1I), *9* (Fig. 1J), *10* (Fig. 1K), and *11* (Fig. 1L) strains. Iron starvation also significantly elevated the virulence of Δ*BbCFEM5* (Fig. 1F) and *7* (Fig. 1H) strains. LT_50_ results showed (Fig. 1M; Table S2) that Δ*BbCFEM1*, *3*, *7*, and *10* showed significantly lower virulence under 0.4 mM BPS conditions than under the condition of appropriate iron. However, there was no significant difference in the virulence between the wild-type strains and the CFEM family knockout strains.

**TABLE 4:** Log-rank tests were performed between the wild-type and mutant strains and individual strain under iron starvation

	Topical infection		Injection infection	
Strains	*χ*^2^ value	*P* value	*χ*^2^ value	*P* value
WT	0.0	0.900	1.3	0.3
Δ*BbCFEM1*	9.3	0.003	88.7	<0.001
Δ*BbCFEM2*	4.4	0.040	62.8	<0.001
Δ*BbCFEM3*	74.7	<0.001	126.0	<0.001
Δ*BbCFEM4*	72.7	<0.001	138.0	<0.001
Δ*BbCFEM5*	10.1	0.002	8.7	0.003
Δ*BbCFEM6*	35.7	<0.001	0.4	0.6
Δ*BbCFEM7*	5.8	0.020	37.8	<0.001
Δ*BbCFEM8*	24.1	<0.001	37.2	<0.001
Δ*BbCFEM9*	147.0	<0.001	15.4	<0.001
Δ*BbCFEM10*	134.0	<0.001	0.2	0.7
Δ*BbCFEM11*	10.7	0.001	181.0	<0.001

### *BbCFEM* gene was required for fungal infection under iron starvation

Interestingly, the mortality rate induced by iron starvation did not exceed 50% in Δ*BbCFEM1*, *3*, and *10* strains. The intrahemocoel injection assay showed that iron starvation did not significantly alter WT strain virulence, similar to topical infection results (Fig. 2A). However, iron starvation significantly lowered the virulence of Δ*BbCFEM1* (Fig. 2B), *2* (Fig. 2C), *3* (Fig. 2D), *4* (Fig. 2E), *8* (Fig. 2I), and *11* (Fig. 2L) strains compared to individual mutant strain. In contrast, iron starvation increased the virulence of Δ*BbCFEM7* (Fig. 2H) and *9* (Fig. 2J) strains compared to individual mutant strains. Results demonstrated that *BbCFEM* genes promote fungal virulence through iron acquisition. LT_50_ results showed (Fig. 2M; Table S2) that Δ*BbCFEM1*, *3*, *4*, *8*, *11* strains had significantly lower virulence under 0.4 mM BPS conditions than under appropriate iron conditions. Compared to moderate iron levels, Δ*BbCFEM5* strains showed significantly higher virulence at 0.4 mM BPS. Notably, the virulence was not significantly different between wild-type strains and other CFEM family gene knockout strains.

### Compensatory effects between *BbCFEM* genes

**Fig 3:**
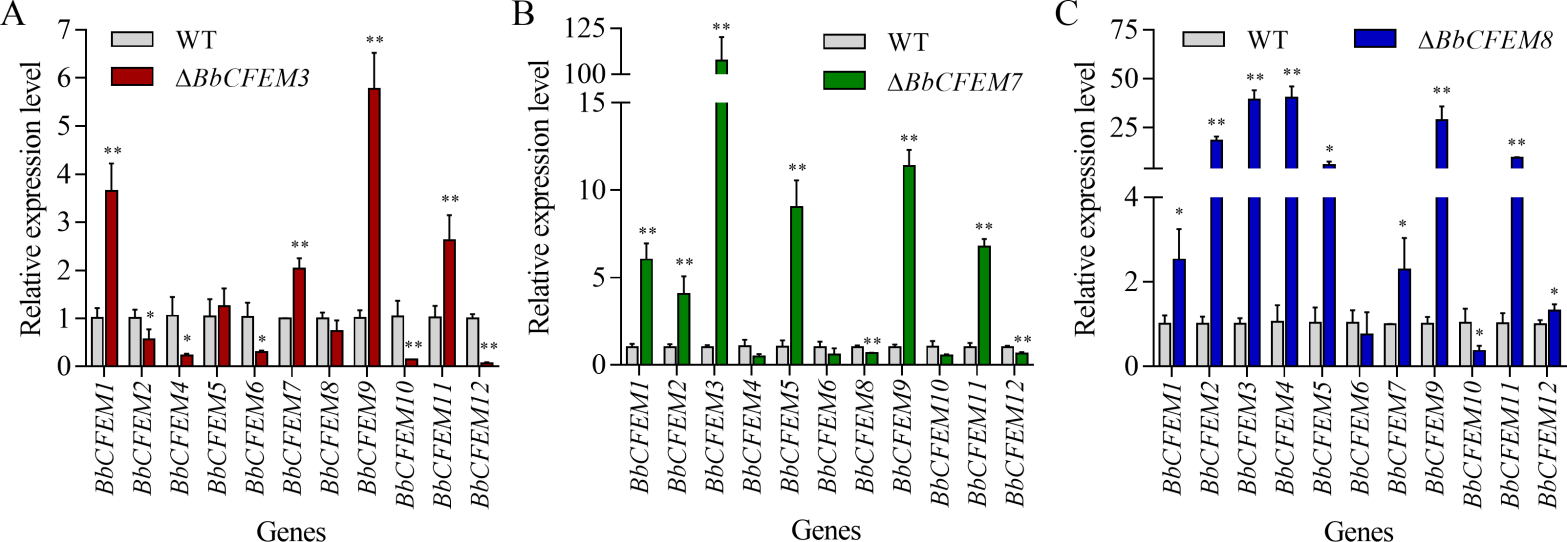
Compensation effect of *CFEM* family genes in *B. bassiana*. (A) In the absence of the *BbCFEM3* gene, *CFEM* family genes compensate for the phenomenon at the transcriptional level in *B. bassiana*. (B) In the absence of the *BbCFEM7* gene, *CFEM* family genes compensate for the phenomenon at the transcriptional level. (C) In the absence of the *BbCFEM8* gene, *CFEM* family genes compensate for the phenomenon at the transcriptional level. Unpaired Student’s *t*-test: *P* < 0.05 (*) and *P* < 0.01 (**); error bars: standard deviation.

Δ*BbCFEM3*, *7*, and *8* strains were selected based on phenotype and virulence data to detect the expression of other *BbCFEM* genes and determine whether there was a compensatory effect between *BbCFEM* genes. *BbCFEM3* gene loss significantly elevated the expression of *BbCFEM1*, *7*, *9*, and *11* genes and significantly reduced the expression of *BbCFEM2*, *4*, *6*, *10*, and *12* genes (Fig. 3A). Furthermore, *BbCFEM7* knockdown significantly increased *BbCFEM1*, *2*, *3*, *5*, *8*, and *11* gene expression and significantly decreased *BbCFEM8* and *12* gene expression (Fig. 3B). In addition, *BbCFEM8* gene loss significantly upregulated *BbCFEM1*, *2*, *3*, *4*, *5*, *7*, *9*, *11*, and *12* and significantly downregulated *BbCFEM10* (Fig. 3C). These findings indicate a compensatory role of *BbCFEM* genes in fungal iron acquisition.

## DISCUSSION

The InterPro database (www.ebi.ac.uk/interpro/) contains over 6,000 CFEM sequences. However, not all CFEM proteins can bind heme (30). This means that the iron obtained by CFEM family proteins may also come from the powerful Fe^2+^/Fe^3+^ besides heme. Meanwhile, there are more potential mechanisms of iron acquisition in the CFEM family. Herein, we report the contribution of 11 CFEM domain proteins of *B. bassiana* to fungal virulence at 0.4 mM BPS via injection infection and body wall infection. Results showed that the virulence contributions of some *CFEM* family genes under severe iron starvation conditions were significantly inconsistent with those under moderate iron conditions, both with increased virulence and with decreased virulence. This suggests that the contribution of *BbCFEM* family genes varies under different iron starvation conditions.

Further comparison analysis showed that (28) (Table 5) the effects of iron starvation through body wall infection were significantly reduced under moderate iron (0 mM BPS), moderate iron hunger (0.2 mM BPS), and severe iron hunger (0.4 mM BPS). The contribution of the same gene to fungal virulence is also different under various iron starvation conditions. For example, *BbCFEM6* showed little independent contribution under moderate iron conditions and significant contribution under both moderate and moderate iron starvation conditions. Particularly, the virulence contribution was significantly superior under moderate iron starvation conditions compared to under severe iron starvation conditions. A similar pattern was observed in other genes. The same results were observed in the blood cavity injection group. For example, the contribution of *BbCFEM11* to fungal virulence increased with increasing levels of iron starvation. The results summarize the division of genes in the *BbCFEM* family at different levels of iron starvation. *BbCFEM7* and *8* contribute to fungal virulence under all conditions. *BbCFEM1*, *2*, *3*, *4*, and *11* can contribute to greater fungal virulence under severe iron starvation. *BbCFEM5*, *6*, *9*, and *10* perform better under mild iron starvation conditions than under severe iron starvation conditions. This study provides the functional distribution of CFEM domain genes in different iron environments. It is well known that the amount of iron in different environments is very different. In pathogenic fungi, the ability to obtain iron in different environments influences their growth and development (28). Our study systematically reports which BbCFEM family genes are active under different iron levels. This result helps to reveal the evolutionary strategies of pathogenic fungi in nature to adapt to the environment and population reproduction. In addition, our results also showed that only two *BbCFEM* genes (*7* and *8*) contributed to fungal virulence under both topical infection and injection infection conditions under moderate iron starvation conditions, while in moderate and severe iron starvation conditions, only two *BbCFEM* genes contributed to fungal virulence. More members of the *BbCFEM* family are activated and exert virulence. This result means that the application of biocontrol fungi such as *B. bassiana* can be properly combined with iron-chelating agents to face different iron environments.

**TABLE 5:** The division of *BbCFEM* family genes in response to iron starvation regulation at different iron starvation levels^*a*^

Iron starvation condition		*BbCFEM* family genes										
		1	2	3	4	5	6	7	8	9	10	11
Topical infection	0 mM BPS	−	−	−	−	−	−	+++	++	−	−	−
	0.2 mM BPS	+	−	−	++	+	++	+++	+++	++	++	++
	0.4 mM BPS	+	+	++	++	−	+	++	+++	++	+++	++
Injection infection	0 mM BPS	−	−	−	−	−	−	+++	++	−	−	−
	0.2 mM BPS	−	−	−	++	++	++	+++	+++	+++	++	++
	0.4 mM BPS	++	++	++	++	−	−	++	++++	−	−	+++

^*a*^ “+” represents the toxic contribution of the gene under this condition, and the number of plus signs is used to distinguish the contribution degree of the same gene in different iron levels. “−” indicates that the gene has no significant contribution in this condition.

Several reports have presented the physiological mechanism of CFEM family genes in recent years. Notably, genes in this domain can acquire iron. For example, MaCFEM protein interactions in *M. anisopliae* with plants occur through competition for iron (9). The *C. albicans* CFEM protein helps the fungus transport heme across cell walls to obtain iron (25, 31). The CFEM protein also participates in the formation of biofilms (18, 19). Herein, Δ*BbCFEM7* and *8* were sensitive to sorbitol stress, suggesting that *BbCFEM7* and *8*, as components of the outer membrane protein, also directly affect the integrity of the cell wall. In addition, Δ*BbCFEM1*, *2*, *3*, *4*, *5*, *6*, *10*, and *11* were sensitive to H_2_O_2_ stress. Furthermore, Δ*BbCFEM3*, *4*, *5*, *6*, *7*, *8*, *10*, and *11* were sensitive to menadione. *BbCFEM7* and *8* were sensitive to NaCl. These results indicate that *BbCFEM* family genes promote iron acquisition, cell wall stress, and oxidative stress, which is consistent with plant and human pathogenic fungi. Importantly, in *M. acridum*’s study of infecting locusts, the absence of *MaCFEM1* led to rapid proliferation of opportunistic bacteria in the insect host’s gut, which quickly led to insect death (10). In this case, *MaCFEM1*’s contribution to the virulence of the fungus was reversed, compared to *B. bassiana*. This result also reflects the differentiation of *CFEM* domain genes for fungal virulence and adaptive evolution to a certain extent.

The members of a gene family often have similar sequences and functions, indicating that gene redundancy can be compensated by other homologous genes (32, 33). A study found that the expression levels of other members may be increased to compensate for the loss of function when members of a gene family are suppressed or inactivated (34, 35). This transcriptional regulatory mechanism does not involve mutual regulation within gene families and may also be regulated by external signals. A similar phenomenon has been reported in the *BbCFEM* family. In this study, *BbCFEM3*, *7*, and *8* knockdowns caused upregulation of RNA expression of other members of the family (Fig. 3). The upregulation of RNA expression can compensate for the damage caused by the deletion of a gene in this family. Redundancy and compensation mechanisms in gene families are key for organism adaptation to different environments. Some gene families show strong compensation ability under environmental stress (temperature, hypoxia, etc.), thus improving the survival rate of organisms.

Fungal species have several gene families, which provide important support for the evolution of species in different ecological niches. Studies have assessed the function of gene families in entomopathogenic fungi, such as *SOD* family and *MARVEL* family (36). Notably, the function of genes in the same family is significantly different. Besides, the reported genes with unknown functions may be useful. In this study, results confirmed that the *BbCFEM* family is involved in the conservation of the iron starvation response pathway in fungi, improving the understanding of the functional division of the fungal gene family.

Nevertheless, there are still shortcomings in this study that we cannot confirm at this stage. For example, we only reported the *CFEM* family domain gene in one strain of *B. bassiana*, which did not further extend the functional differentiation and compensation phenomenon of this family gene to more strains to verify. Furthermore, the compensation phenomenon of *BbCFEM* family genes in this study is worthy of being verified at the protein expression level, and the compensation mechanism is also worthy of further exploration in different fungal species.

In summary, this study reports functional differentiation and compensation in *BbCFEM* genes under varying iron conditions. This family gene helps fungi to compete and acquire biological iron in saprophytic environment, insect surface infection stage, insect blood cavity proliferation, and saprophytic stage through compensation and rational division of labor in an iron-starved environment, thus contributing to the differentiation and virulence of fungi. Therefore, this study may provide a new theoretical basis for the specificity and imbalance of virulence of entomopathogenic fungi when they infect multiple hosts. However, further studies should reveal the mechanism by which the *BbCFEM* family accomplishes this compensation mechanism.

### Conclusion

The *CFEM* family genes (1–11) of *B. bassiana* contribute differently in response to different carbon and nitrogen sources, growth resistance to chemical stressors, and virulence. Furthermore, with the exception of *BbCFEM7* and *BbCFEM8*, most *CFEM* domain genes did not play a critical role in vegetative growth. It was also observed that most genes influenced the response of fungi to cell wall stress and different iron starvation conditions following body wall infection and injection infection. In addition, the deletion of a single gene in the *BbCFEM* family leads to increased upregulation of other members of the family, indicating the presence of functional compensation mechanisms between *BbCFEM* gene families. This study provides new insights into the genetic families of entomopathogenic fungi and the mechanisms by which they infect pests.

## Data Availability

All data presented in this study are available in the article.

## SUPPLEMENTAL MATERIAL

Supplemental material spectrum.03096-24-s0001.pdf Tables S1 and S2.

ASM does not own the copyrights to Supplemental Material that may be linked to, or accessed through, an article. The authors have granted ASM a non-exclusive, world-wide license to publish the Supplemental Material files. Please contact the corresponding author directly for reuse.
