# Supplementary material for: The virulence contribution of the CFEM family genes of Beauveria bassiana is closely influenced by the external iron environment
Source: Microbiol Spectr. 2025 Mar 21;13(5):e03096-24. doi: 10.1128/spectrum.03096-24 (PMC12054149; doi:10.1128/spectrum.03096-24)
Supplement: Supplemental material — Tables S1 and S2. [file spectrum.03096-24-s0001.pdf]

Supplementary information

Supplementary Table 1. qRT-PCR primers used in this study.

Genes	Gene ID	Forward (5'-3')	Reverse (5'-3')
BbCFEM1	BBA_02419	ATATCAGCACTGACGGCTCG	AAGAGACTGCGTGGGAACAG
BbCFEM2	BBA_02928	CAAATGCGCCTATGAGTGCC	CGCAGCCTTTTTCACAGCTT
BbCFEM3	BBA_05711	CGAATCGACATGCTCTCCGA	GCAACCTCCCATTGATCCCA
BbCFEM4	BBA_06341	TGCACGAGAATCAACCTGCT	TCGAGCTCATATGCCGTGTC
BbCFEM5	BBA_06531	AACCCCCTCAACGACATTCC	GCAATACTGTTGCCGCTGAG
BbCFEM6	BBA_06784	GCAAGTCGGAGAACCAGGAA	CAACTTGCAAGACGGTTCGG
BbCFEM7	BBA_07758	AGAAATCGCAAGTTCGGGGT	AGATGTGGCTCTGAGTTGCC
BbCFEM8	BBA_08327	TGCTTCAACAACATGCGTGG	GGTGATGATGTGGTTGGGCT
BbCFEM9	BBA_08376	CACCTCCGGCTACCACAAAT	CCTCAGGGGTAATGGGGAGA
BbCFEM10	BBA_08726	TTGCATCTGCGAGACCAACT	AGAGGCACAAATCATGCCGA
BbCFEM11	BBA_09290	TGGTATTAGCTCCCTGCCCT	CATGCAGTCAGTTTCGACGC
BbCFEM12	BBA_09339	GTGTCATTCAGTCTTGCGGC	GTCTGGTTGTTGACGGGAGT
β-actin		GGCAACATTGTCATGTCTGG	TTTGCTGGAAGGTGGATAGG

Supplementary Table 2. Effects of iron starvation on LT_{50} through topical and injection infection.

	Topical infection			Injection infection		
Strains	0 mM BPS	0.2 Mm BPS	0.4 Mm BPS	0 mM BPS	0.2 Mm BPS	0.4 Mm BPS
WT	5.69 ± 0.18	5.80 ± 0.29	5.81 ± 0.26	3.52 ± 0.21	3.35 ± 0.04	3.29 ± 0.03
Δ*BbCFEM1*	6.59 ± 0.28^{b}	8.59 ± 0.88^{a}	-	3.55 ± 0.10^{b}	3.38 ± 0.05^{b}	4.88 ± 0.51^{a}
Δ*BbCFEM2*	5.61 ± 0.27	6.01 ± 0.63	6.29 ± 0.43	3.47 ± 0.04^{b}	3.38 ± 0.09b^{b}	4.41 ± 0.79^{a}
Δ*BbCFEM3*	5.24 ± 0.26	5.80 ± 0.17	-	3.47 ± 0.11^{b}	3.49 ± 0.36^{b}	4.78 ± 0.52^{a}
Δ*BbCFEM4*	5.56 ± 0.14	6.50 ± 0.19	6.58 ± 0.91	3.25 ± 0.06^{b}	4.54 ± 0.24^{a}	4.63 ± 0.44^{a}
Δ*BbCFEM5*	5.95 ± 0.40	6.81 ± 0.22	5.94 ± 0.47	3.82 ± 0.19^{b}	6.84 ± 0.49^{a}	3.18 ± 0.04^{b}
Δ*BbCFEM6*	6.00 ± 0.31^{b}	8.39 ± 1.11^{a}	6.75 ± 0.76^{b}	3.85 ± 0.09^{b}	5.33 ± 0.42^{a}	3.60 ± 0.18^{b}
Δ*BbCFEM7*	-	-	7.02 ± 0.69	5.26 ± 0.30^{a}	5.82 ± 0.14^{a}	4.18 ± 0.18^{b}
Δ*BbCFEM8*	-	-	-	5.45 ± 0.17^{b}	6.01 ± 0.34^{ab}	6.72 ± 0.54^{a}
Δ*BbCFEM9*	5.79 ± 0.20^{b}	7.57 ± 0.31^{a}	6.51 ± 0.35^{ab}	4.03 ± 0.21^{b}	7.23 ± 0.05^{a}	3.58 ± 0.20^{b}
Δ*BbCFEM10*	5.61 ± 0.38	6.52 ± 0.78	-	3.70 ± 0.10^{b}	4.59 ± 0.28^{a}	3.65 ± 0.12^{b}
Δ*BbCFEM11*	5.95 ± 0.45^{b}	7.46 ± 0.43^{a}	6.74 ± 0.43^{ab}	3.47 ± 0.15^{c}	4.91 ± 0.25^{b}	5.73 ± 0.57^{a}

Note: Ane-way analysis of variance followed by a Tukey's post hoc test were used to compare among multiple groups. $p < 0.05$ was considered statistically significant.
